# Efficacy and safety of epcoritamab in Japanese patients with relapsed or refractory diffuse large B-cell lymphoma: 3-year follow-up from the EPCORE NHL-3 trial

**DOI:** 10.1007/s10147-025-02788-0

**Published:** 2025-05-28

**Authors:** Koji Izutsu, Takahiro Kumode, Junichiro Yuda, Hirokazu Nagai, Yuko Mishima, Youko Suehiro, Kazuhito Yamamoto, Tomoaki Fujisaki, Kenji Ishitsuka, Kenichi Ishizawa, Takayuki Ikezoe, Momoko Nishikori, Daigo Akahane, Jiro Fujita, Pegah Jafarinasabian, David Soong, Barbara D’Angelo Månsson, Ami Takahashi, Elena Favaro, Noriko Fukuhara

**Affiliations:** 1 Department of Hematology, National Cancer Center Hospital, Tsukiji 5-1-1, Chuo-ku, Tokyo, 104-0045 Japan; 2 Department of Hematology and Rheumatology, Kindai University, Osaka, Japan; 3 Departments of Hematology and Experimental Therapeutics, Office for the Promotion of Hematological Treatment Development, National Cancer Center Hospital East, Kashiwa, Japan; 4 Department of Hematology, National Hospital Organization Nagoya Medical Center, Nagoya, Japan; 5 Department of Hematology Oncology, Cancer Institute Hospital, Japanese Foundation for Cancer Research, Tokyo, Japan; 6 Department of Hematology and Cell Therapy, National Hospital Organization Kyushu Cancer Center, Fukuoka, Japan; 7 Department of Hematology and Cell Therapy, Aichi Cancer Center, Nagoya, Japan; 8 Department of Hematology, Japan Red Cross Society, Matsuyama Red Cross Hospital, Matsuyama, Japan; 9 Department of Hematology and Rheumatology, Kagoshima University, Kagoshima, Japan; 10 Third Department of Internal Medicine, Yamagata University, Yamagata, Japan; 11 Department of Hematology, Fukushima Medical University Hospital, Fukushima, Japan; 12 Department of Hematology, Graduate School of Medicine, Kyoto University, Kyoto, Japan; 13 Department of Hematology, Tokyo Medical University, Tokyo, Japan; 14 Department of Hematology and Oncology, Graduate School of Medicine, Osaka University, Osaka, Japan; 15 AbbVie, North Chicago, IL USA; 16 Genmab, Plainsboro, NJ USA; 17 Genmab, Copenhagen, Denmark; 18 Genmab, Tokyo, Japan; 19 Department of Hematology, Tohoku University, Sendai, Japan

**Keywords:** B-cell lymphoma, Non-Hodgkin lymphoma, Bispecific antibodies, Clinical trial

## Abstract

**Background:**

Primary results from the EPCORE NHL-3 trial (NCT04542824) showed deep, durable responses in Japanese patients with relapsed or refractory (R/R) diffuse large B-cell lymphoma (DLBCL) treated with single-agent epcoritamab, a subcutaneous CD3xCD20 bispecific antibody. Here, we report 3-year follow-up of safety and efficacy.

**Methods:**

Japanese patients with R/R CD20^+^ DLBCL and  ≥ 2 prior systemic therapies received epcoritamab (0.16/0.8-mg step-up doses, then 48-mg full doses) according to the approved label. The primary endpoint was overall response rate per independent review committee.

**Results:**

As of July 12, 2024, 36 patients received epcoritamab (median follow-up, 36.7 months). Overall/complete response rates were 56%/47%. Median duration of response was 15.2 months. Median duration of complete response was not reached; an estimated 53% of complete responders remained in complete response at 3 years. Median progression-free/overall survival (PFS/OS) were 4.1/14.9 months overall; neither was reached among complete responders. Three-year PFS/OS estimates were 25%/39% overall and 53%/71% in complete responders. Among 30 evaluable patients, 17 (57%) became minimal residual disease (MRD) negative, which was associated with longer PFS (cycle 3 day 1 landmark analysis). The most common treatment-emergent adverse events (TEAEs) were cytokine release syndrome (83%), injection-site reaction (69%), and neutropenia (39%), consistent with previous reports. No fatal TEAEs occurred.

**Conclusions:**

With  > 3 years of follow-up, epcoritamab treatment has consistently shown durable responses and high rates of MRD negativity in Japanese patients with R/R DLBCL. Safety was similar to previous reports. These long-term remissions reaffirm encouraging outcomes with epcoritamab for this challenging-to-treat population.

**Supplementary Information:**

The online version contains supplementary material available at 10.1007/s10147-025-02788-0.

## Introduction

Diffuse large B-cell lymphoma (DLBCL), the most common B-cell non-Hodgkin lymphoma (B-NHL), comprises 36% of all malignant lymphomas and about half of all B-cell neoplasms in Japan [1, 2]. For patients who remain event free for 2 years after initial treatment, long-term outcomes are encouraging, with survival rates comparable to the general population [3, 4]. However, patients in third-line or later treatment with relapsed or refractory (R/R) disease are challenging to treat and have a poor prognosis, with a median overall survival (OS) of < 7 months [5, 6].

While there have been advances in targeted therapies in this setting, novel, accessible, tolerable, and efficacious treatments are needed to improve long-term outcomes for patients with R/R DLBCL. Polatuzumab vedotin and chimeric antigen receptor (CAR) T-cell therapies are approved in Japan for the treatment of DLBCL; however, efficacy and/or accessibility remain suboptimal [7–10]. CAR T-cell therapies are not readily accessible due to their restriction to specialized centers and lengthy manufacturing process [11, 12]. T-cell-engaging bispecific antibodies, an emerging treatment class, have demonstrated encouraging antitumor activity in R/R DLBCL [13–16].

Epcoritamab is the first and only off-the-shelf subcutaneous CD3xCD20 bispecific antibody approved for different types of R/R LBCL, including DLBCL, after ≥ 2 systemic therapy lines in various geographies, including Japan, the US, and Europe [17–19]. In Japan, epcoritamab is approved for adults with R/R DLBCL, high-grade B-cell lymphoma, primary mediastinal large B-cell lymphoma, and, recently, follicular lymphoma [17]. Long-term results from the pivotal global EPCORE NHL-1 trial (median follow-up, 37.1 months) and primary results from the EPCORE NHL-3 trial (median follow-up, 8.4 months; conducted in Japan) have demonstrated deep, durable responses with manageable safety in patients with R/R DLBCL treated with epcoritamab monotherapy [20, 21]. Here, we report long-term efficacy and safety results with over 3 years of follow-up among patients with R/R DLBCL from the DLBCL expansion cohort (phase 2) of the Japanese EPCORE NHL-3 trial.

## Materials and methods

### Study design and patients

The EPCORE NHL-3 trial (ClinicalTrials.gov identifier: NCT04542824; JapicCTI-205408; jRCT2080225312) has been described previously [20]. In brief, patients aged ≥ 20 years with documented R/R CD20^+^ DLBCL, measurable disease, ≥ 2 prior systemic therapy lines, including ≥ 1 anti-CD20–containing regimen, and ineligibility for or previous failure of autologous stem cell transplant (ASCT) received epcoritamab monotherapy (0.16- and 0.8-mg step-up doses on cycle 1 day 1 and 8, respectively, then 48-mg full doses) in 28-day cycles: weekly in cycles 1–3, every 2 weeks in cycles 4–9, and every 4 weeks in cycles 10 and beyond until unacceptable toxicity or disease progression. Details on cytokine release syndrome (CRS) prophylaxis have been previously published [20]. Hospitalization was required for 24 hours following administration of the first full dose of epcoritamab on cycle 1 day 15.

### Endpoints and assessments

The primary endpoint was overall response rate (ORR) per independent review committee (IRC) assessment according to Lugano classification [22]. Protocol-specified secondary endpoints included complete response (CR) rate, time to response, time to CR, duration of response (DOR), duration of CR (DOCR), progression-free survival (PFS), OS, and safety. Immunoglobulin levels were assessed at screening and day 1 of each cycle. Minimal residual disease (MRD) was assessed in circulating tumor DNA (ctDNA; clonoSEQ next-generation sequencing assay, Adaptive Biotechnologies). MRD-negative patients were defined as having undetectable ctDNA (0 copies of ctDNA detected) in ≥ 1 on-treatment sample; all other MRD-evaluable patients were considered MRD positive, as previously described [20]. A landmark analysis was conducted for PFS and OS by MRD-negativity status up to cycle 3 day 1. The landmark analysis included MRD-evaluable patients who, by cycle 3 day 1, had neither a PFS event nor death. Patients were stratified by MRD status and followed from cycle 3 day 1 until a PFS event or death occurred.

### Statistical analysis

The full analysis and safety analysis sets, each comprising patients who received ≥ 1 epcoritamab dose, were used for all efficacy and safety assessments. The MRD-evaluable set, comprising patients in the full analysis set with ≥ 1 on-treatment MRD sample, was used for MRD assessments. Efficacy endpoints were assessed in predefined subgroups, including by response and MRD-negativity status.

ORR was defined as the proportion of patients with a best overall response of CR or partial response (PR). CR rate was defined as a best overall response of CR. Where applicable, 95% confidence intervals (CIs) were calculated using the Clopper–Pearson method. Efficacy endpoints were determined per positron emission tomography–computed tomography (PET-CT) according to Lugano criteria and IRC [22]. Continuous data were summarized with descriptive statistics; categorical data were summarized with frequency count and 95% CI.

Time to response was defined as time from cycle 1 day 1 to CR or PR, and time to CR was defined as time from cycle 1 day 1 to CR. DOR was defined as time from first CR or PR to documented disease progression or death, and DOCR was defined as time from first CR to documented disease progression or death. PFS was defined as the time from cycle 1 day 1 to documented disease progression or death. OS was defined as the time from cycle 1 day 1 to death. Time-to-event endpoints, including DOR, DOCR, PFS, and OS, were summarized using the Kaplan–Meier method with survival analysis at prespecified timepoints.

### Ethics

An independent ethics committee approved the protocol prior to trial initiation. The trial was conducted in compliance with the International Council for Harmonisation Good Clinical Practice (GCP) E6(R2) guidelines, local guidelines (i.e., Japan GCP), principles of the Declaration of Helsinki, and applicable regulatory requirements. Informed consent documents were reviewed and signed by all patients prior to trial enrollment.

## Results

### Study population and exposure

As of July 12, 2024, 36 patients had received ≥ 1 dose of epcoritamab. Baseline demographic and disease characteristics have been reported previously [20]. In this challenging-to-treat, highly refractory population, median age was 68.5 years (range 44–89), 6 patients (16.7%) had transformed DLBCL, 28 (77.8%) had Ann Arbor stage III or IV disease, and 21 (58.3%) had an International Prognostic Index of 3–5 (Table 1)﻿. Patients had a median of 3 prior lines of therapy (range 2–8); 30.6% of patients had ≥ 4 prior lines of therapy, 55.6% had primary refractory disease, 80.6% had double-refractory disease (i.e., refractory to both anti-CD20 and an alkylating agent, regardless of the treatments being used in the same or different treatment lines), and 58.3% were refractory to ≥ 2 consecutive lines of therapy. A total of 5 patients (13.9%) received prior bendamustine.

**Table 1:** Demographic and baseline clinical characteristics for Japanese adults from the overall population with relapsed or refractory diffuse large B-cell lymphoma

Characteristic	Overall, *N* = 36
Median age, years (range)	68.5 (44–89)
Age, years, *n* (%)	
< 65	10 (27.8)
65 to < 75	18 (50.0)
≥ 75	8 (22.2)
Sex at birth, *n* (%)	
Male	17 (47.2)
Female	19 (52.8)
ECOG performance status, *n* (%)	
0	21 (58.3)
1	13 (36.1)
2	2 (5.6)
DLBCL type, *n* (%)	
De novo	30 (83.3)
Transformed from follicular lymphoma	6 (16.7)
DLBCL cell-of-origin classification per IHC, *n* (%)	
Germinal center B-cell	8 (22.2)
Non–germinal center B-cell	16 (44.4)
Unknown/missing	11 (30.6)
Not done	1 (2.8)
Ann Arbor stage, *n* (%)	
I or II	8 (22.2)
III or IV	28 (77.8)
IPI, *n* (%)	
0–2	15 (41.7)
3–5	21 (58.3)
Median time from end of last antilymphoma therapy to first dose, months (range)	2.8 (0.1–39.3)
Median number of prior lines of therapy (range)	3 (2–8)
Primary refractory disease, *n* (%)	20 (55.6)
Double-refractory disease, *n* (%)^a^	29 (80.6)
Refractory to last systemic therapy, *n* (%)	29 (80.6)
Refractory to ≥ 2 consecutive lines of therapy, *n* (%)	21 (58.3)
Prior autologous stem cell transplant, *n* (%)	7 (19.4)

*DLBCL* diffuse large B-cell lymphoma, *ECOG* Eastern Cooperative Oncology Group, *IHC* immunohistochemistry, *IPI* International Prognostic Index

^a^Refractory to both anti-CD20 and an alkylating agent regardless of the 2 treatments being in the same or different treatment lines

Patients initiated a median of 5.5 epcoritamab cycles (range 1–41) and the median duration of treatment was 5 months (range 0–42). At data cutoff, 7 patients (19.4%) were on treatment and 29 (80.6%) had discontinued treatment. The primary reasons for treatment discontinuation were disease progression (63.9%), adverse events (AEs; 13.9%), and patient request (2.8%).

### Efficacy

With a median follow-up of 36.7 months (range 1.5+ to 41.7), the ORR was 55.6%, with a CR rate of 47.2% per IRC (Table 2). Median times to response and CR were 1.4 and 2.7 months, respectively (Table 3), aligned with scheduled response assessments (first assessment at 6 weeks, second assessment at 12 weeks). Deepening of responses was observed with 1 patient converting from PR to CR between the primary analysis and current assessment (at the 24-week imaging assessment). ORR/CR rates were consistently high across subgroups, including patients aged ≥ 75 years (75.0%/62.5%), patients with Ann Arbor stage III or IV disease (53.6%/42.9%), patients with 2, 3, or ≥ 4 prior therapies (56.3%/50.0%, 66.7%/55.6%, and 45.5%/36.4%), and patients with prior ASCT (71.4%/71.4%; Supplemental Fig. 1). Patients with primary refractory disease had lower ORR/CR rates (35.0%/20.0%) compared with other high-risk subgroups and patients without primary refractory disease (81.3%/81.3%). Among 5 patients with prior bendamustine, 4 (80.0%) had a response.

**Table 2:** Best overall response among Japanese adults from the overall population with relapsed or refractory diffuse large B-cell lymphoma according to independent review committee per Lugano criteria

Best overall response	Overall, *N* = 36
Overall response, *n* (%) [95% CI]	20 (55.6) [38.1–72.1]
Complete response	17 (47.2) [30.4–64.5]
Partial response	3 (8.3) [1.8–22.5]
Stable disease, *n* (%)	2 (5.6)
Progressive disease, *n* (%)	14 (38.9)

*CI* confidence interval

**Table 3:** Efficacy endpoints from the overall population of Japanese adults with relapsed or refractory diffuse large B-cell lymphoma and among patients with CR according to independent review committee per Lugano criteria

	Overall, *N* = 36	Patients with CR, *n* = 17
Median time to response, months (range)	1.4 (1.1–2.6)	1.4 (1.1–2.6)
Median time to CR, months (range)	2.7 (1.1–5.5)	2.7 (1.1–5.5)
Median DOR, months (range) [95% CI]	15.2 (2.5–37.6+) [4.2–NR]	NR (2.5–37.6+) [4.2–NR]
Estimates, %		
2 years	45.0	52.9
3 years	45.0	52.9
Median DOCR, months (range) [95% CI]	NR (1.2–36.2+) [2.7–NR]	NR (1.2–36.2+) [2.7–NR]
Estimates, %		
2 years	52.9	52.9
3 years	52.9	52.9
Median PFS, months (range) [95% CI]	4.1 (0.5–38.9+) [1.2–14.8]	NR (3.9–38.9+) [5.3–NR]
Estimates, %		
2 years	25.0	52.9
3 years	25.0	52.9
Median OS, months (range) [95% CI]	14.9 (1.5–41.7+) [8.4–NR]	NR (11.0–41.7+) [15.0–NR]
Estimates, %		
2 years	41.7	70.6
3 years	38.9	70.6

Estimates are based on the Kaplan–Meier method

*CI* confidence interval, *CR* complete response, *DOCR* duration of complete response, *DOR* duration of response, *NR* not reached, *OS* overall survival, *PFS* progression-free survival

**Fig. 1:**
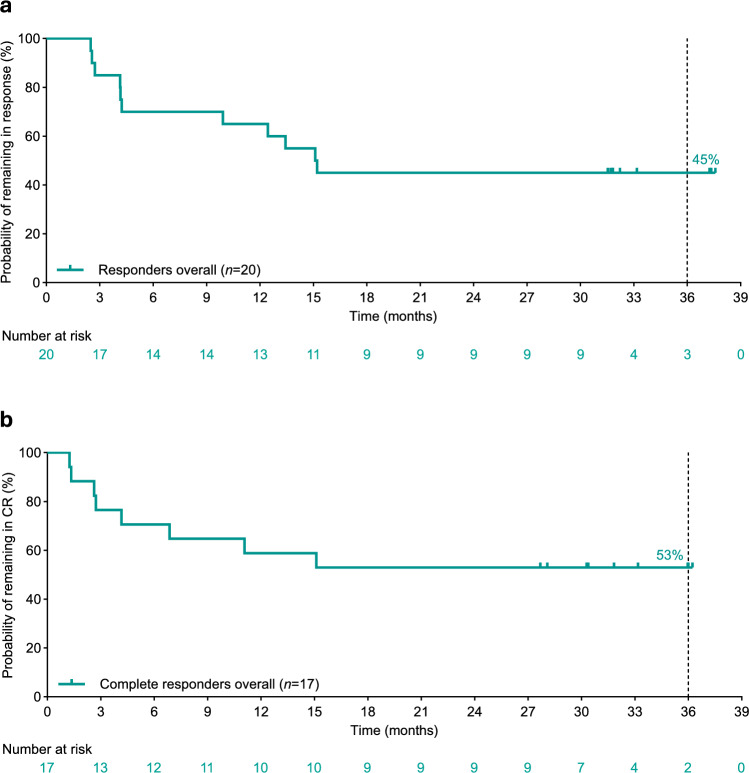
Kaplan–Meier curves for DOR (**a**) and DOCR (**b**) per independent review committee assessment among Japanese adults with relapsed or refractory diffuse large B-cell lymphoma. *CR* complete response, *DOCR* duration of complete response, *DOR* duration of response

**Fig. 2:**
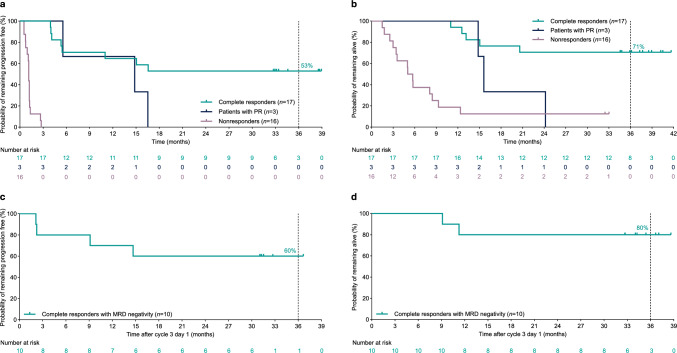
Kaplan–Meier curves for PFS by response (**a**), OS by response (**b**), PFS in complete responders with MRD negativity (**c**), and OS in complete responders with MRD negativity (**d**) among Japanese adults with relapsed or refractory diffuse large B-cell lymphoma. PFS and response are per independent review committee assessment. MRD-negativity status was assessed in a cycle 3 day 1 landmark analysis. The landmark analysis included MRD-evaluable patients who, by cycle 3 day 1, had neither a PFS event nor death. Patients were stratified by MRD status and followed from cycle 3 day 1 until PFS event or death. *MRD* minimal residual disease, *OS* overall survival, *PFS* progression-free survival, *PR* partial response

The median DOR and DOCR were 15.2 months and not reached, respectively, based on Kaplan–Meier estimates (Fig. 1a, b). At 3 years, an estimated 45.0% of responders remained in response and an estimated 52.9% of complete responders remained in CR (Table 3). As expected, an association between depth of response and long-term outcomes was observed (Fig. 2a, b). Median PFS was 4.1 months and median OS was 14.9 months. In patients who had a CR, median PFS and OS were not reached. At 3 years, an estimated 52.9% of patients with CR remained progression free and 70.6% remained alive (Table 3).

Among 30 MRD-evaluable patients, 56.7% became MRD negative. Median duration of MRD negativity was 11.6 months (range 0.0+ to 13.0). A cycle 3 day 1 landmark analysis of 15 MRD-evaluable patients, 11 (73.3%) of whom were MRD negative, revealed longer PFS in patients with MRD negativity (median, not reached) versus patients without MRD negativity (median, 7.8 months after cycle 3 day 1); 3-year estimates for PFS were 54.5% and 25.0% for patients with MRD negativity and without MRD negativity, respectively. OS was also improved in patients with MRD negativity by cycle 3 day 1 (median, not reached; 3-year estimate, 72.7%) versus patients without MRD negativity by cycle 3 day 1 (median, 7.0 months after cycle 3 day 1; 3-year estimate, 25.0%). Among complete responders with MRD negativity, 3-year estimates for PFS and OS were 60.0% and 80.0% (Fig. 2c, d).

Although no patients had prior CAR T-cell therapy, 4 patients went on to receive subsequent CAR T-cell therapy after epcoritamab (median of 154 days from last epcoritamab dose [range 56–327]). Three of these 4 patients remained alive at data cutoff, although 2 patients progressed after subsequent CAR T-cell therapy. No patients received subsequent ASCT; 1 patient received subsequent allogeneic stem cell transplant.

### Safety

The most common treatment-emergent AEs (TEAEs) were CRS (83.3%); injection-site reaction, including injection-site reaction and erythema (69.4%); neutropenia, including decreased neutrophil count and neutropenia (38.9%); hypokalemia (27.8%); lymphopenia, including decreased lymphocyte count and lymphopenia (27.8%); decreased appetite (25.0%); thrombocytopenia, including decreased platelet count and thrombocytopenia (25.0%); leukopenia, including decreased white blood cell count and leukopenia (22.2%); and rash (22.2%; TEAE overview shown in Table 4). Overall, 50.0% of patients had grade 1 CRS events, 25.0% had grade 2, and 8.3% had grade 3; median time to onset was 15.5 days, median time to resolution was 4.5 days, and no events led to epcoritamab discontinuation.

**Table 4:** Overview of treatment-emergent adverse events in Japanese adults with relapsed or refractory diffuse large B-cell lymphoma

	Any grade, *n* (%)	Grade ≥ 3, *n* (%)
Any TEAE	36 (100.0)	30 (83.3)
Serious TEAE	19 (52.8)	14 (38.9)
TEAE leading to treatment discontinuation	5 (13.9)	3 (8.3)
TEAE in ≥ 15% of patients		
Cytokine release syndrome^a^	30 (83.3)	3 (8.3)
Injection-site reaction^b^	25 (69.4)	0
Infections^c^	21 (58.3)	12 (33.3)
Neutropenia^d^	14 (38.9)	14 (38.9)
Hypokalemia	10 (27.8)	6 (16.7)
Lymphopenia^e^	10 (27.8)	9 (25.0)
Decreased appetite	9 (25.0)	0
Thrombocytopenia^f^	9 (25.0)	6 (16.7)
Leukopenia^g^	8 (22.2)	5 (13.9)
Rash	8 (22.2)	1 (2.8)
Malaise	7 (19.4)	0
Nausea	7 (19.4)	0
Constipation	6 (16.7)	0
COVID-19^h^	6 (16.7)	6 (16.7)
Diarrhea	6 (16.7)	0
TEAEs of special interest		
Cytokine release syndrome^a^	30 (83.3)	3 (8.3)
Immune effector cell–associated neurotoxicity syndrome	1 (2.8)	0
Clinical tumor lysis syndrome	0	0

*TEAE* treatment-emergent adverse event

^a^Cytokine release syndrome is shown twice

^b^Injection-site reaction includes injection-site reaction and erythema

^c^Infections and infestations by System Organ Class

^d^Neutropenia includes decreased neutrophil count and neutropenia

^e^Lymphopenia includes decreased lymphocyte count and lymphopenia

^f^Thrombocytopenia includes decreased platelet count and thrombocytopenia

^g^Leukopenia includes decreased white blood cell count and leukopenia

^h^COVID-19 includes COVID-19, COVID-19 pneumonia, and post-acute COVID-19 syndrome

Infections and infestations by System Organ Class occurred in 58.3% of patients. Six patients (16.7%) experienced COVID-19 and 3 patients (8.3%) experienced cytomegalovirus infection. There were no fungal infections. Immunoglobulin levels decreased from baseline after initiation of treatment and remained generally stable through cycle 36 (Supplemental Fig. 2). A total of 15 patients (41.7%) were given concomitant immunoglobulins.

Grade 3 or 4 infections occurred in 33.3% of patients at any timepoint, the most frequent of which were COVID-19, COVID-19 pneumonia, pneumonia, and urinary tract infection, in 8.3% each. Infections occurred in 33.3% of patients in the first 12 weeks (grade ≥ 3 infections, 11.1%) and in 16.7–42.9% of patients in subsequent 12-week intervals between weeks 109 and 144 (range for grade ≥ 3 infections, 14.3–16.7%). Beyond week 108, the most common type of infection was COVID-19 (14.3–28.6% between weeks 109 and 144). Overall, 11 patients (30.6%) experienced serious infections, 4 of whom had 2 events and 1 of whom had ≥ 4 events; 8.3% of patients had a serious infection in the first 12 weeks versus 14.3–16.7% of patients in subsequent 12-week intervals between weeks 109 and 144.

Cytopenias occurred in 25 patients (69.4%), including 3 patients who had 2 events, 5 who had 3 events, and 15 who had ≥ 4 events. A total of 52.8% of patients experienced cytopenias in the first 12 weeks versus 14.3–33.3% of patients in subsequent 12-week intervals between weeks 109 and 144. Cytopenias with ≥ 15% incidence are shown in Table 4. Febrile neutropenia occurred in 1 patient (2.8%; grade 3) and resolved in 5 days. Eleven of 14 patients with neutropenia (78.6%) required granulocyte colony-stimulating factor treatment for neutropenia. Nine patients (25.0%) had thrombocytopenia, 5 (55.6%) of whom required treatment.

TEAEs led to treatment discontinuation in 5 patients (13.9%): COVID-19, chronic myelomonocytic leukemia, muscular weakness, pancreatic carcinoma, and prolonged electrocardiogram QT in 1 patient each. Of those who discontinued treatment due to TEAEs, the median duration of epcoritamab treatment was 11.1 months; median DOR and DOCR were 13.4 months and 11.1 months, respectively. The median DOCR after treatment discontinuation due to TEAEs was 9.8 months.

There were 5 complete responders who had an epcoritamab pause for > 6 weeks. Reasons for dose delay were AEs in all cases. Among these patients, the median duration of treatment pause was 7.6 weeks (range 6–44) prior to restarting treatment. All patients maintained their CR after resuming epcoritamab.

No fatal TEAEs were reported. Rates and severity of CRS, immune effector cell–associated neurotoxicity syndrome (ICANS), and clinical tumor lysis syndrome (CTLS) were unchanged since the primary analysis [20]. CRS mostly followed the first full dose, with 71.4% of patients experiencing a CRS event during that dosing period.

## Discussion

With over 3 years of follow-up, epcoritamab monotherapy continues to yield deep and durable responses in Japanese patients with R/R DLBCL from the EPCORE NHL-3 trial. Overall, patients were high risk, heavily pretreated, and difficult to treat, representative of the real-world population; 77.8% had Ann Arbor stage III or IV disease, the median number of prior treatments was 3, 55.6% had primary refractory disease, and 80.6% had double-refractory disease. An association between depth of response and long-term outcomes was observed. Patients who had CR had median CR duration of not reached, with an estimated 52.9% and 70.6% of complete responders remaining progression free and alive, respectively, at 3 years. Kaplan–Meier curves for PFS and OS among patients with CR appeared to plateau (median PFS and OS, not reached), underscoring that CR translated to encouraging long-term outcomes and a potential for cure in a subset of patients. PFS and OS were improved in patients with versus without MRD negativity (median PFS and OS in MRD-negative patients, not reached). These results reaffirm data from the primary EPCORE NHL-3 analysis [20] and are consistent with the findings from the global trial, EPCORE NHL-1, which recently also reported highly encouraging long-term results with over 3 years of follow-up [21].

With a longer median follow-up of 36.7 months compared with 8.4 months in the primary analysis [20], ORR remained the same, and 1 patient showed deepening of response from PR to CR. Median DOR was not reached overall or among complete responders in the primary analysis [20]; it was also not reached among complete responders with longer follow-up. Consistent with the primary analysis [20], > 3 years of follow-up reaffirmed improved PFS and OS in complete responders compared with the overall population. The long-term safety findings were generally consistent with previous reports, with CRS, infections, injection-site reactions, and neutropenia being the most common TEAEs. No fatal TEAEs have been reported to date. Rates and severity of AEs of special interest (i.e., CRS, ICANS, and CTLS) were unchanged. Long-term follow-up demonstrated that incidences of infections and serious infections were generally consistent over time. These results underscore that epcoritamab drives long-term benefit with a potential to cure a subset of patients with DLBCL.

Bispecific antibodies are gaining traction as a treatment for adults with LBCL [23]. The observed deep and durable responses (ORR: 55.6%; CR rate: 47.2%; among patients who had a CR, median DOCR, PFS, and OS were not reached) were comparable or favorable to global data for other bispecific antibodies, though intertrial comparisons should be interpreted cautiously due to varying study designs and patient backgrounds. In the challenging-to-treat R/R setting, ORRs and CR rates with other bispecific antibody therapies range from 52% to 66% and from 32% to 50%, respectively [15, 16, 24]. Primary refractory disease remains a major challenge, as reflected by the lower response rates in this study (ORR/CR rate: 35.0%/20.0%) compared with patients without primary refractory disease (ORR/CR rate: 81.3%/81.3%). Nevertheless, in the larger, global EPCORE NHL-1 trial, 32% of patients with primary refractory disease had a CR (vs 53% of patients without primary refractory disease) [14], suggesting the potential role of epcoritamab in this difficult-to-treat population. Our reports with over 3 years of follow-up highlight the long-term efficacy and safety of epcoritamab.

Although﻿ CAR T-cell therapies have shown relatively high response rates in global studies (ORR rates: 52–82%; CR rates: 40–54%) [9, 25, 26], access to therapy, delays caused by the need for transfer to treatment facilities and bridging therapy, manufacturing time, risk of production failure, and high incidence of CRS and ICANS make them a less-than-ideal option for patients with later-line R/R DLBCL, for whom fast disease control is needed [11, 12]. Epcoritamab induced efficacy in Japanese patients comparable with CAR T-cell therapies (epcoritamab median DOR, 15.2 months vs median DOR with CAR T-cell therapies, 5.6–9.1 months; epcoritamab median PFS, 4.1 months vs median PFS with CAR T-cell therapies, ~ 6 months; epcoritamab median OS, 14.9 months vs median OS with lisocabtagene maraleucel, 14.7 months) [27, 28]. Epcoritamab is also a more convenient, off-the-shelf treatment that allows rapid T-cell engagement and B-cell killing [29] without the need for prior bridging or debulking therapy.

The EPCORE NHL-3 trial is limited by its open-label, single-country study design and enrollment during the COVID-19 pandemic. Results are comparable to the global EPCORE NHL-1 trial, which enrolled a more diverse population [14].

## Conclusion

In conclusion, long-term follow-up of over 3 years from the EPCORE NHL-3 trial reaffirmed that epcoritamab leads to durable responses and high rates of MRD negativity in Japanese patients with R/R DLBCL. Safety was manageable and consistent with prior findings; no new safety signals were observed. With over half of patients with CR remaining in remission at 3 years, these data suggest long-term disease-free survival, providing a promising treatment option for Japanese patients with R/R DLBCL.

## Supplementary Information

Below is the link to the electronic supplementary material.

Supplementary file1 (PDF 633 KB)

## Data Availability

Deidentified individual participant data collected during the trial will not be available upon request for further analyses by external independent researchers. Aggregated clinical trial data from the trial are provided via publicly accessible study registries/databases as required by law. For more information, please contact ClinicalTrials@genmab.com.
