# Supplementary material for: Efficacy and safety of epcoritamab in Japanese patients with relapsed or refractory diffuse large B-cell lymphoma: 3-year follow-up from the EPCORE NHL-3 trial
Source: Int J Clin Oncol. 2025 May 28;30(8):1631–40. doi: 10.1007/s10147-025-02788-0 (PMC12296760; doi:10.1007/s10147-025-02788-0)

SUPPLEMENTAL MATERIALS

Efficacy and safety of epcoritamab in Japanese patients with relapsed or refractory diffuse large B-cell lymphoma: 3-year follow-up from the EPCORE NHL-3 trial

Authors:

Koji Izutsu, MD, PhD,[1] Takahiro Kumode, MD, PhD,[2] Junichiro Yuda, MD, PhD,[3] Hirokazu Nagai, MD, PhD,[4] Yuko Mishima, MD, PhD,[5] Youko Suehiro, MD, PhD,[6] Kazuhito Yamamoto, MD, PhD,[7] Tomoaki Fujisaki, MD,[8] Kenji Ishitsuka, MD, PhD,[9] Kenichi Ishizawa, MD, PhD,[10] Takayuki Ikezoe, MD, PhD,[11] Momoko Nishikori, MD, PhD,[12] Daigo Akahane, MD, PhD,[13] Jiro Fujita, MD, PhD,[14] Pegah Jafarinasabian, MD, PhD,[15] David Soong, PhD,[16] Barbara D'Angelo Månsson, PhD,[17] Ami Takahashi, PhD,[18] Elena Favaro, MD, PhD,[17] Noriko Fukuhara, MD, PhD[19]

[1]Department of Hematology, National Cancer Center Hospital, Tokyo, Japan; [2]Department of Hematology and Rheumatology, Kindai University, Osaka, Japan; [3]Departments of Hematology and Experimental Therapeutics, Office for the Promotion of Hematological Treatment Development, National Cancer Center Hospital East, Kashiwa, Japan; [4]Department of Hematology, National Hospital Organization Nagoya Medical Center, Nagoya, Japan; [5]Department of Hematology Oncology, Cancer Institute Hospital, Japanese Foundation for Cancer Research, Tokyo, Japan; [6]Department of Hematology and Cell Therapy, National Hospital Organization Kyushu Cancer Center, Fukuoka, Japan; [7]Department of Hematology and Cell Therapy, Aichi Cancer Center, Nagoya, Japan; [8]Department of Hematology, Japan Red Cross Society, Matsuyama Red Cross Hospital, Matsuyama, Japan; [9]Department of Hematology and Rheumatology, Kagoshima University, Kagoshima, Japan; [10]Third Department of Internal Medicine, Yamagata University, Yamagata, Japan; [11]Department of Hematology, Fukushima Medical University Hospital, Fukushima, Japan; [12]Department of Hematology, Graduate School of Medicine, Kyoto University, Kyoto, Japan; [13]Department of Hematology, Tokyo Medical University, Tokyo, Japan; [14]Department of Hematology and Oncology, Graduate School of Medicine, Osaka University, Osaka,

Japan; [15]AbbVie, North Chicago, IL, USA; [16]Genmab, Plainsboro, NJ, USA; [17]Genmab, Copenhagen, Denmark; [18]Genmab, Tokyo, Japan; [19]Department of Hematology, Tohoku University, Sendai, Japan

SUPPLEMENTAL RESULTS

Supplemental Fig. 1 ORRs (a) and CR rates (b) by independent review committee assessment in subgroups of Japanese adults with relapsed or refractory diffuse large B-cell lymphoma. ASCT, autologous stem cell transplant; CI, confidence interval; CR, complete response; DLBCL, diffuse large B-cell lymphoma; ECOG, Eastern Cooperative Oncology Group; ORR, overall response rate; pLOT, prior lines of therapy; y, years.

a

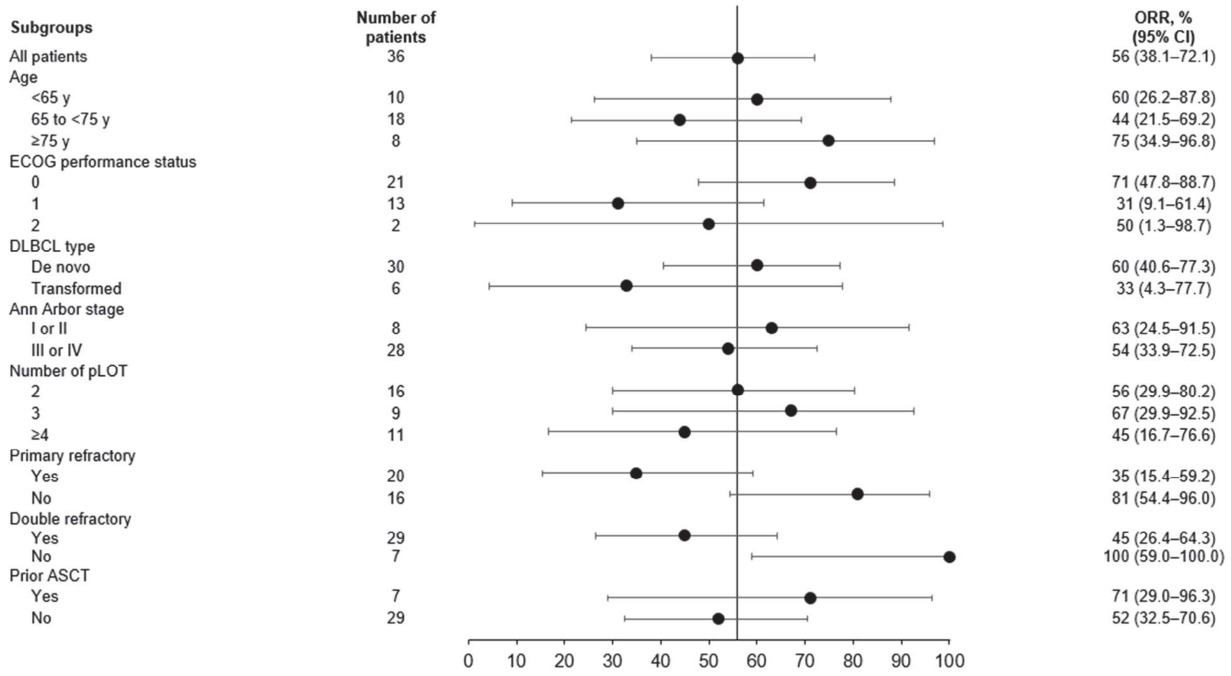

b

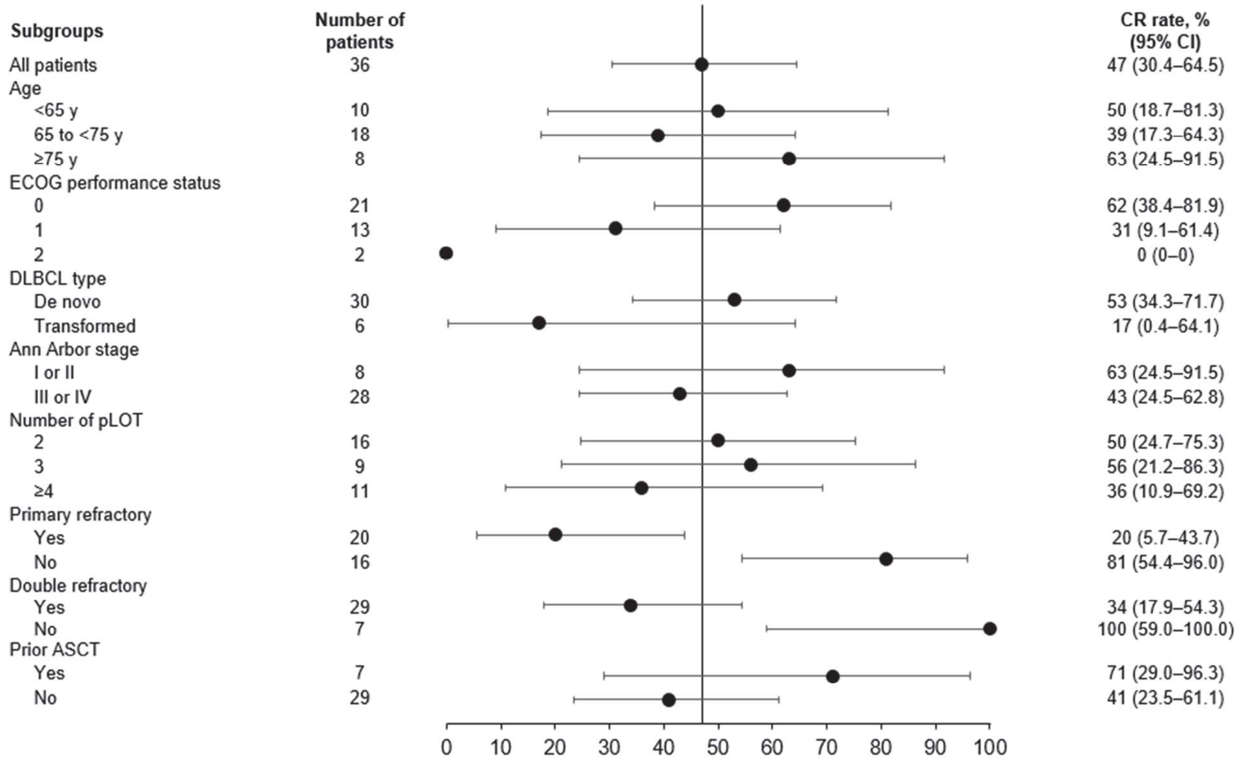
Subgroups
Number of patients
CR rate, % (95% CI)
All patients 36 47 (30.4–64.5)
Age
<65 y 10 50 (18.7–81.3)
65 to <75 y 18 39 (17.3–64.3)
≥75 y 8 63 (24.5–91.5)
ECOG performance status
0 21 62 (38.4–81.9)
1 13 31 (9.1–61.4)
2 2 0 (0–0)
DLBCL type
De novo 30 53 (34.3–71.7)
Transformed 6 17 (0.4–64.1)
Ann Arbor stage
I or II 8 63 (24.5–91.5)
III or IV 28 43 (24.5–62.8)
Number of pLOT
2 16 50 (24.7–75.3)
3 9 56 (21.2–86.3)
≥4 11 36 (10.9–69.2)
Primary refractory
Yes 20 20 (5.7–43.7)
No 16 81 (54.4–96.0)
Double refractory
Yes 29 34 (17.9–54.3)
No 7 100 (59.0–100.0)
Prior ASCT
Yes 7 71 (29.0–96.3)
No 29 41 (23.5–61.1)
0 10 20 30 40 50 60 70 80 90 100

Supplemental Fig. 2 Mean change from baseline in immunoglobulin G levels over time among Japanese adults with relapsed or refractory diffuse large B-cell lymphoma. C, cycle; D, day.

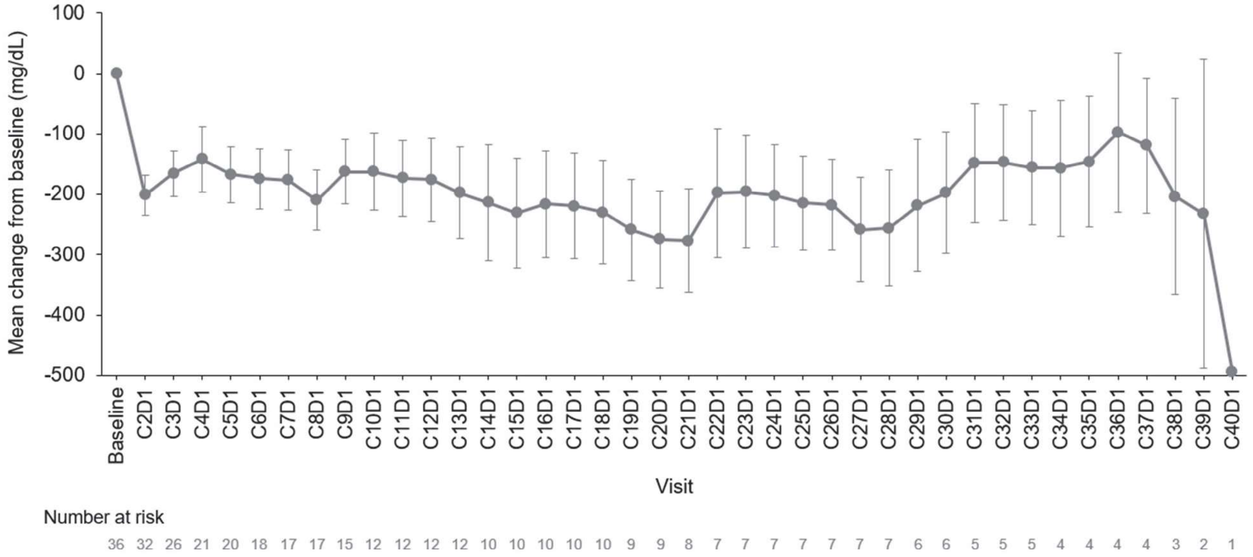

Supplement: Supplementary file 1 — Supplementary file1 (PDF 633 KB) [file 10147_2025_2788_MOESM1_ESM.pdf]
